# Mo-doped boron nitride monolayer as a promising single-atom electrocatalyst for CO_2_ conversion

**DOI:** 10.3762/bjnano.10.55

**Published:** 2019-02-22

**Authors:** Qianyi Cui, Gangqiang Qin, Weihua Wang, Lixiang Sun, Aijun Du, Qiao Sun

**Affiliations:** 1 State Key Laboratory of Radiation Medicine and Protection, Collaborative Innovation Center of Radiation Medicine of Jiangsu Higher Education Institutions, School for Radiological and Interdisciplinary Sciences, Soochow University, Suzhou 215123, China; 2 School of Chemistry and Chemical Engineering, Qufu Normal University, Qufu, Shandong, 273165, China; 3 College of Chemistry and Materials Science, Ludong University, Yantai 264025, China; 4 School of Chemistry, Physics and Mechanical Engineering, Queensland University of Technology, Brisbane, QLD 4001, Australia

**Keywords:** boron nitride monolayer, CO_2_ conversion, density functional theory, single-atom electrocatalyst

## Abstract

The design of new, efficient catalysts for the conversion of CO_2_ to useful fuels under mild conditions is urgent in order to reduce greenhouse gas emissions and alleviate the energy crisis. In this work, a series of transition metals (TMs), including Sc to Zn, Mo, Ru, Rh, Pd and Ag, supported on a boron nitride (BN) monolayer with boron vacancies, were investigated as electrocatalysts for the CO_2_ reduction reaction (CRR) using comprehensive density functional theory (DFT) calculations. The results demonstrate that a single-Mo-atom-doped boron nitride (Mo-doped BN) monolayer possesses excellent performance for converting CO_2_ to CH_4_ with a relatively low limiting potential of −0.45 V, which is lower than most catalysts for the selective production of CH_4_ as found in both theoretical and experimental studies. In addition, the formation of OCHO on the Mo-doped BN monolayer in the early hydrogenation steps is found to be spontaneous, which is distinct from the conventional catalysts. Mo, as a non-noble element, presents excellent catalytic performance with coordination to the BN monolayer, and is thus a promising transition metal for catalyzing CRR. This work not only provides insight into the mechanism of CRR on the single-atom catalyst (Mo-doped BN monolayer) at the atomic level, but also offers guidance in the search for appropriate earth-abundant TMs as electrochemical catalysts for the efficient conversion of CO_2_ to useful fuels under ambient conditions.

## Introduction

In the past decades, considerable carbon dioxide emissions into the atmosphere due to large-scale anthropogenic industrial manufacturing have resulted in global climate change effects [1]. Thus, it is very important to develop advanced technologies for efficient CO_2_ capture, storage and conversion [2]. Carbon dioxide storage technologies have made great progress in recent years [3–5], which provides a feasible foundation for converting CO_2_ into useful fuel and commodity chemicals [6]. For example, CO_2_ can be converted to methane, methanol and formic acid, and all of which can be used as energy sources and chemical materials at the global scale [7–11]. In this sense, the CO_2_ reduction reaction (CRR) by electrochemical methods is promising because it can take place at room temperature and atmospheric pressure with useful catalysts, making it feasible for extensive application and integration [12–14].

Metal nanoparticles supported on various substrates have been extensively investigated as heterogeneous catalysts in many reactions [10,15–17]. Over the past few decades, researchers have focused on decreasing the size of metal nanoparticles in order to improve the surface area/volume ratio of low-coordinated metal atoms in order to enhance the selectivity toward specific species and to improve the electrocatalytic performance [18–20]. The ultimate size limit for metal particles is single-atom catalysts (SACs), in which the isolated single metal atoms distribute on the substrates in an ordered fashion [21]. Moreover, the single metal atoms are supported on the substrates as active sites, which exhibit higher catalytic efficiency than conventional nanoparticles [22–25]. To date, the catalysts that have employed various single transition metal (TM) atoms anchored on the different substrates such as graphene [26–29] and graphitic carbon nitride [30–34], have presented good performance and high efficiency.

As an analogue of graphene, boron nitride (BN) nanomaterials have sparked worldwide interest in exploring their applications in many fields, both experimentally and theoretically, due to their excellent properties, such as high chemical stability, thermal conductivity, oxidation resistance and refractory nature [11,35–41]. Moreover, BN nanomaterials have been used as superior substrates for doping various transition metals by electron beam irradiation [42] or solvent exfoliation [43] to form selected point defects, which are preferred to growing specific boron vacancies [42,44]. Recent reports show that single-TM-doped BN nanomaterials have been used as efficient catalysts in the reactions of N_2_ fixation and CO oxidation [45–46]. It is worth noting that Chen and co-workers reported that single Mo supported on defective BN nanosheets presents a highly efficient electrocatalyst for nitrogen fixation with a very small overpotential of only 0.19 V. In addition, through molecular dynamics modeling, they demonstrated that Mo-doped BN synthesized in acidic conditions is stable at high temperature (500 K) [45]. In our previous reports, we have studied BN nanomaterials used as efficient materials for CO_2_ capture and gas separation [35,47]. The excellent performance of BN nanomaterials in various applications have inspired us to study whether the materials can be efficient catalysts for CO_2_ reduction. To answer this question, we have screened possible SACs involving fifteen TMs (TM = Sc to Zn, Mo, Rh, Ru, Pd and Ag) anchored on the boron vacancy in a BN monolayer as electrocatalysts for CO_2_ conversion through comprehensive density functional theory (DFT) calculations. Based on the calculated results, single Mo doped onto a BN (Mo-doped BN) monolayer was selected as the catalyst for further investigation of CO_2_ conversion due to its high selectivity and activation for CO_2_. The study shows that Mo-doped BN monolayers can be used as a promising catalyst for CO_2_ reduction to CH_4_ with a low limiting potential of −0.45 V. More importantly, Mo is an abundant element in the earth, thus using Mo-doped BN monolayers as an electrocatalyst for CO_2_ conversion can significantly reduce the cost compared with conventional noble-metal catalysts, such Au, Ag, Pt, Pd and so on [30,33,48–49]. This work provides insight and guidance to experimentalists in search of low cost, high efficiency SACs for converting CO_2_ to useful hydrocarbon fuels.

## Results and Discussion

### Transition metal selection for CO_2_ reduction reaction

**Figure 1:**
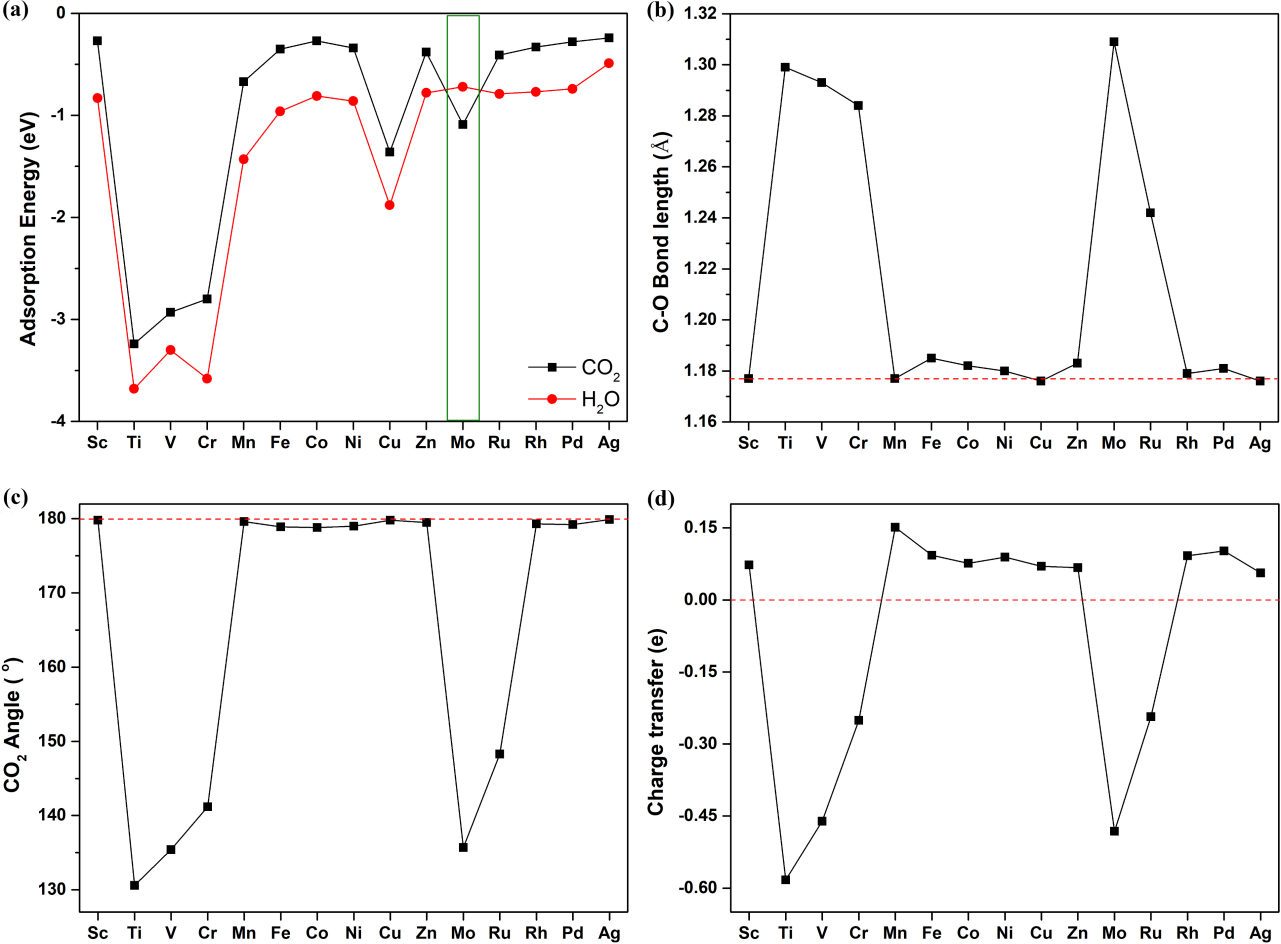
(a) Adsorption energy of CO_2_ and H_2_O on various single transition metal (TM) doped BN monolayers. (b) The length of the C–O bond of CO_2_ that interacts with the TM atom doped onto the BN monolayers. (c) The angle of the captured CO_2_ molecule. (d) The charge transfer of CO_2_ adsorbed on various catalysts. The red dashed lines represent values corresponding to the free CO_2_ molecule in Figure 1(b), (c) and (d).

For efficient CO_2_ reduction, the most critical requirement is that the CO_2_ molecule can selectively adsorb onto the catalyst and guarantee sufficient activation for CRR [9,11]. This is a widely used, basic criterion for selecting catalyst materials for electrocatalysts in TM-doped BN for the N_2_ reduction reaction, whereby the criterion is that the catalyst can facilitate the chemisorption of N_2_ molecules [45]. To screen for promising transition metals to be doped onto BN monolayers (TM-doped BN) as the SACs, the interaction between CO_2_ and various TM (Sc to Zn, Mo, Ru, Rh, Pd and Ag) doped BN monolayers were considered. Moreover, for efficient reduction of CO_2_, the interaction between CO_2_ and the catalyst should be stronger than that between H_2_O and the catalyst. Therefore, we calculated the adsorption energy (Δ*E*_ads_) of CO_2_ and H_2_O on TM-doped BN monolayers. The Δ*E*_ads_ of all structures is calculated using Δ*E*_ads_ = *E*_gas–catal_ − *E*_gas_ − *E*_catal_, where *E*_gas–catal_ represents the energy of the whole absorbed structure, and *E*_gas_ and *E*_catal_ represent the energy of the free gas molecule and the clean surface, respectively. The calculation results are exhibited in Figure 1 and Supporting Information File 1, Table S2, showing various properties of the most stable configurations of CO_2_ and H_2_O adsorbed on TM-doped BN (Figure 1a) and corresponding structural parameters, such as C–O bond length (Figure 1b) and the angle (Figure 1c) of the CO_2_ molecule adsorbed onto the TM-doped BN. As shown in Figure 1a, there are significant differences in the CO_2_ adsorption on the various TM-doped BN monolayers, where the system with the more negative value of adsorption energy means a stronger interaction. Therefore, it can be seen clearly from Figure 1a that there is weak adsorption between CO_2_ and some of the TM-doped BN monolayers, including Sc, Mn, Fe, Co, Ni, Zn, Ru, Rh, Pd and Ag, whose adsorption energy values are not negative enough to satisfy the requirements as catalysts for CRR.

In contrast with Figure 1a, the geometric configuration of CO_2_ captured by TM-doped BN is in good agreement with the values of the adsorption energies – Figure 1b reflects that the inert C–O double bond of CO_2_ can be stretched by strong adsorption on some TM-doped BNs (Ti, V, Cr and Mo). On the other hand, the variation of the C–O bond is negligible which has weak adsorption on the TMs (Sc, Mn, Fe and so on) mentioned above (Figure 1b). In addition, it is worth noting that the C–O bond interacts with Mo, whose tensile effect is the most significant in the series of TMs we screened. Meanwhile, the CO_2_ angle is also determined by the strength of the interaction of CO_2_ with different TMs that anchor on the BN monolayer. The configuration of captured CO_2_ is significantly distorted by strong chemisorption as illustrated in Figure 1a–c. Regarding the electronic properties, physisorption of CO_2_ results in a positive charge state; however, chemisorption of CO_2_ results in a negative charge state, because electrons are transferred to the captured CO_2_ from the TM-doped BN monolayer (Figure 1d).

**Figure 2:**
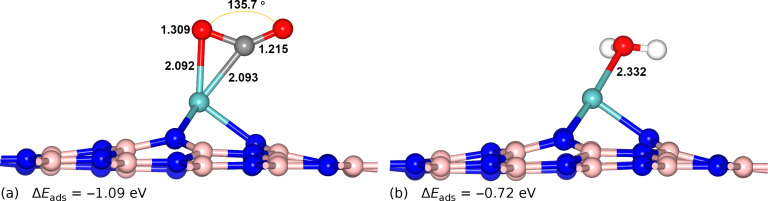
The most stable, optimized configurations of CO_2_ (a) and H_2_O (b) adsorption on Mo-doped BN monolayers. The distances are labeled in units of Å. The C, O, H, Mo, B and N atoms in the configuration are colored as gray, red, white, cyan, pink and blue, respectively.

CO_2_ must be readily captured by the catalyst, and the interaction between CO_2_ and the catalyst should be stronger than that between H_2_O and the catalyst. From Figure 1a we can also see that, except for the Mo-doped BN monolayer, whose interaction with CO_2_ is stronger than that of H_2_O, the other TM-doped BNs prefer adsorbing H_2_O to CO_2_. The most stable configurations of CO_2_ and H_2_O adsorption on the surface of the Mo-doped BN monolayer are shown in Figure 2a and b, respectively. Figure 2a shows that the two chemical bonds, C–Mo and O–Mo, formed with lengths of 2.093 Å and 2.092 Å, respectively. The angle of O–C–O is stretched from 180° for the isolated CO_2_ to 135.7° for the captured one. The O–C bond interacted with Mo atom lengths from 1.176 Å of the free CO_2_ molecule to 1.309 Å of the adsorbed one (Supporting Information File 1, Table S3). The obvious distortions in the geometric configuration are in agreement with the strong chemisorption with an adsorption energy of −1.09 eV, which means that the CO_2_ is activated by the Mo-doped BN monolayer. The strong adsorption is also supported by a large value (0.482 e^−^) of electron transfer from the catalyst to the CO_2_ molecule. However, for the interaction of H_2_O on the Mo-doped BN monolayer, the changes in the geometric structure of the H_2_O molecule are different from the adsorbed CO_2_ (Figure 2b and Supporting Information File 1, Table S4) and the adsorption energy of H_2_O on the surface of the catalyst is –0.72 eV. This demonstrates that CO_2_ not only strongly interacts with and is activated on the Mo-doped BN monolayer but is also preferable to H_2_O. As the projected density of states (Supporting Information File 1, Figure S3) shows, we note that there is an overlapping of the p- and d-orbitals of the CO_2_ absorbed structure located at around −1.36/−0.46 eV. This is the result of the strong interaction between CO_2_ and Mo-doped BN monolayer. For the H_2_O adsorbed on the catalyst, the change in the electronic structure is less obvious than for CO_2_. This is in good agreement with the observed interaction between CO_2_ and the Mo-doped BN monolayer, which is stronger than that of H_2_O with the catalyst. Therefore, the Mo-doped BN monolayer was chosen as the SAC for the further investigation.

### CO_2_ electrocatalytic reduction

**Figure 3:**
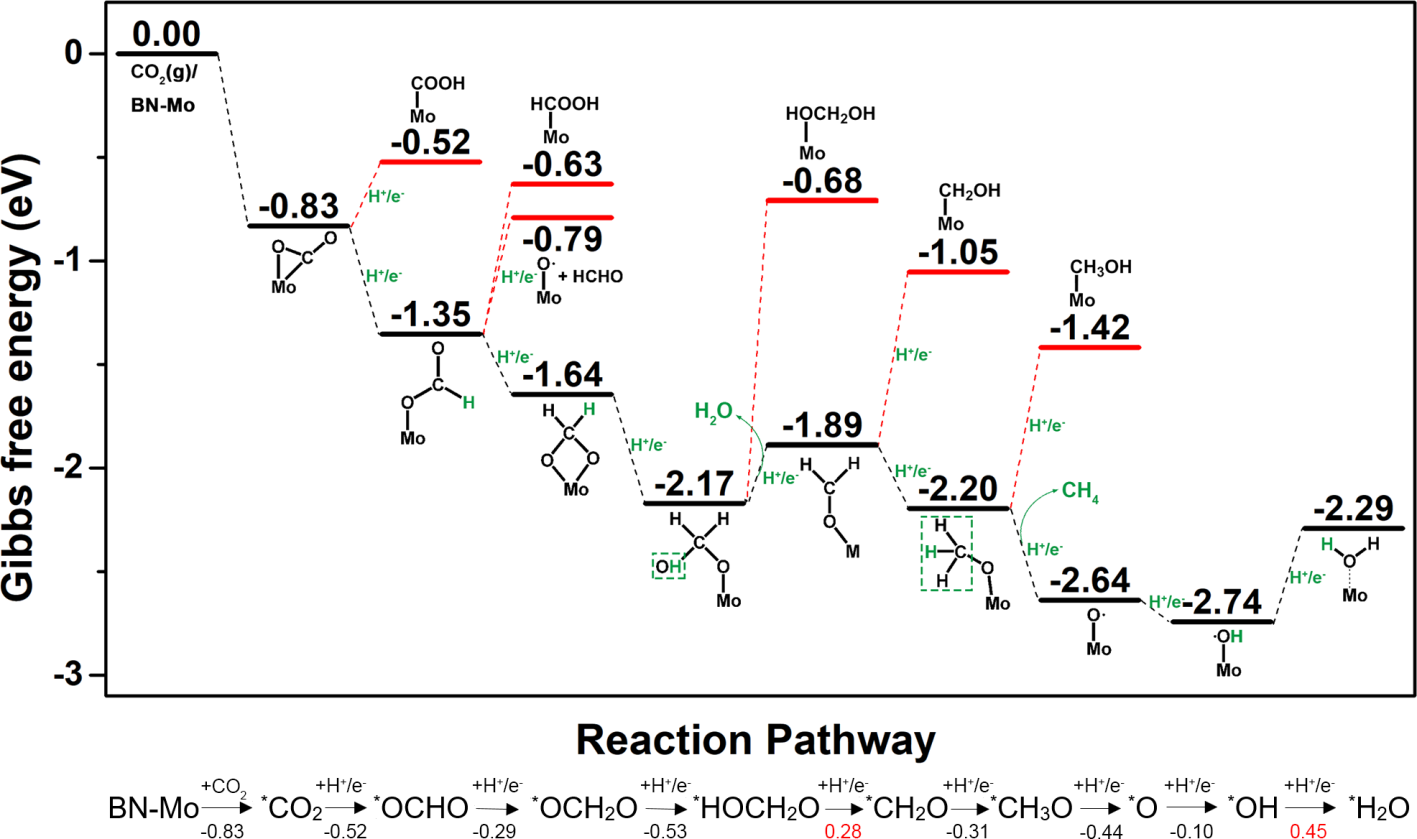
The profile of the Gibbs free energy. A standardized isolated CO_2_ molecule and a clean Mo-doped BN monolayer are taken as zero, the minimum free energy pathway for CO_2_ conversion into CH_4_ and H_2_O on a Mo-doped BN monolayer. The values of the formation energy intermediates are labelled in units of eV (top). At the bottom, the reaction energies are given in black and the red numbers indicate spontaneous and nonspontaneous reactions in eV, respectively. The asterisk (*) represents intermediates chemisorbed on the Mo-doped BN monolayer.

**Figure 4:**
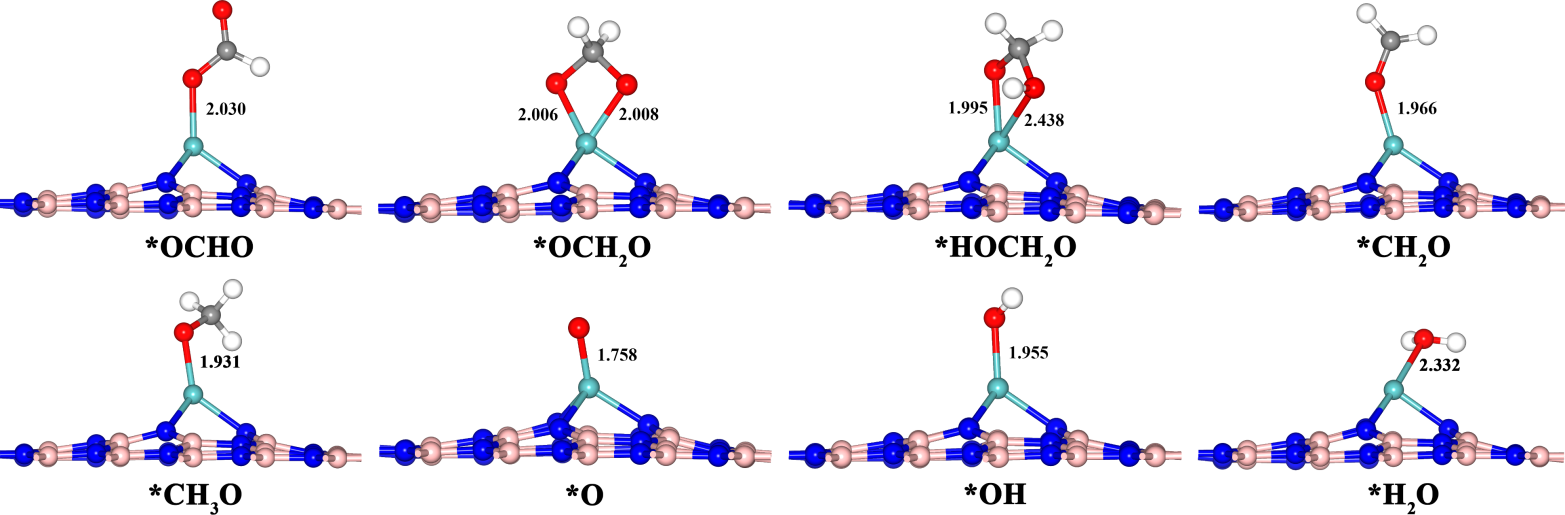
Optimized geometric configurations of the various intermediates with the lowest energy in each step along the reaction pathway of CRR on a Mo-doped BN monolayer. The selected distances are labelled in units of Å. The C, O, H, Mo, B and N atoms in the configuration are colored as gray, red, white, cyan, pink and blue, respectively. The asterisk (*) represents the chemisorbed species.

The reaction mechanism of Mo-doped BN monolayer as a SAC for CRR was investigated via DFT calculations. The profile of the Gibbs free energy of the possible intermediates at each hydrogenation step is shown in Figure 3 and the free energy variations of the main intermediates are displayed in the flow chart at the bottom. The detailed data of Figure 3 are given in Supporting Information File 1, Table S5. We have considered all possible intermediates and the optimized structures of the main product with the lowest energy in each step along the CRR pathways shown in Figure 4. The configurations of the by products are displayed in Supporting Information File 1, Figure S1. As shown in Figure 3, the total Gibbs free energy of the isolated CO_2_ molecule and the Mo-doped BN monolayer is defined as zero. For the first step of CO_2_ hydrogenation, the possible intermediates involve *OCHO and *COOH. According to the calculated results, the reduction of CO_2_ to *OCHO (Δ*G* = −1.35 eV) is exothermic with a value of 0.52 eV, while the formation of *COOH (Δ*G* = −0.52 eV) is an endothermic reaction of about 0.31 eV. These results imply that the early hydrogenation steps may take place at the C atom of the captured CO_2_ rather than at the terminal O atom. The following step involves three competitive intermediates, including *OCH_2_O, *HCOOH and CH_2_O where the *O atom interacts with the surface of the SACs. Obviously, illustrated in as Figure 4, the evolution of *OCH_2_O (Δ*G* = −1.64 eV) in the second step is accompanied by an energy release of 0.29 eV. The production of CH_2_O from the surface of the catalyst (Δ*G* = −0.79 eV) demands an energy input of 0.56 eV. The energy input for *HCOOH is even higher than that of C_2_HO, whose value is 0.72 eV. Therefore the formation of *OCH_2_O dominates in the second step. The products of the first two steps are agreement with the hypothesis above that the C atom (rather than the O atom) is a proton accepting site at the initial reduction reaction stage of the system. Then the third H^+^/e^−^ interacts with one of the O atoms that bonds with Mo atom and products *HOCH_2_O (Δ*G* = −2.17 eV). The reaction is exothermic with a value of 0.53 eV. The fourth H^+^/e^−^ results from the same O atom of the third step, converting into a *CH_2_O molecule (Δ*G* = −1.89 eV) with dissociation of a H_2_O molecule whose reaction is endothermic with a value of 0.28 eV. The competitive product is *HOCH_2_OH (Δ*G* = −0.68 eV), where H^+^/e^−^ bonds with the O atom at the other side, and the energy input (1.49 eV) is too high to reach. The fifth step, the formation of *CH_3_O (Δ*G* = −2.20 eV), is exothermic with a value of 0.31 eV. By contrast, the production of *CH_2_OH is an endothermic reaction, which demands 0.84 eV energy input. The sixth H^+^/e^−^ is obtained by the *CH_3_O, forming CH_3_OH (Δ*G* = −1.42 eV) or CH_4_ (Δ*G* = −2.64 eV) which are desorbed from the catalyst. The feasible reaction mechanism of this step is the production of CH_4_ with an energy release of 0.44 eV, while the formation process of CH_3_OH requires an energy input of 0.78 eV. As CH_4_ has formed and desorbed from the surface, there is only a single O atom to bond with the Mo atom. Therefore, the successive hydrogenation reaction steps are the H^+^/e^−^ interaction with the O atom to form *OH (Δ*G* = −2.74 eV) and *H_2_O (Δ*G* = −2.29 eV). The seventh reaction step, the formation of *OH, occurs with an energy release of 0.10 eV, while the energy input for the last step of H^+^/e^−^ interacts with *OH to form H_2_O occurs at a relatively high value of 0.45 eV.

From the above analysis, we can explicitly determine that the Mo-doped BN monolayer exhibits high activation and selectivity as an electrocatalyst for CO_2_ reduction to CH_4_ along the whole reaction pathway. Moreover, the value of the energy input is also noteworthy. To sum up, the rate-determining step of CRR is the last hydrogenation step, *H_2_O production with an energy input 0.45 eV for CO_2_ reduction to CH_4_. According to the computational hydrogen electrode (CHE) model, the limiting potential (*U*_lim_) is defined as: *U*_lim_ *=* −*∆G*_max_*/e*, where *∆G*_max_ is the largest free energy in CRR. Therefore, the limiting potential of CRR on the surface of the Mo-doped BN monolayer is −0.45 V. A less negative value corresponds to a lower energy input. Moreover, the Gibbs free energy diagram of the HER on the surface of the Mo-doped BN monolayer is shown in Figure S2 in Supporting Information File 1. We clearly see that the limiting potential of CRR and HER on the Mo-doped BN is −0.45 V and −0.62 V, respectively. Therefore, we can draw the conclusion that the CRR is predominant on the catalyst due to its relatively low energy demand. The limiting potential is relatively low among most of the catalysts to selectively produce CH_4_ for CRR with the potential in the range of −0.3 to −1.0 V, involving noble metal and non-noble metals, both theoretically and experimentally. According to previous research, the application of non-noble metals as catalysts for CO_2_ reduction to CH_4_ could overcome the high cost of noble metal catalysts, however, the energy demand is relatively high. For instance, transition-metal carbides have been extensively researched. The energy cost of CRR on the surface of Mo_2_C [10] and WC [50] is 0.56 and 1.00 eV, respectively. Similarly, Sun et al. reported that the energy input of Cr_3_C_2_ and Mo_3_C_2_ catalyzed in the conversion of CO_2_ to CH_4_ is 0.64 and 0.77 eV [9]. In addition, the performance of Mo-doped BN is comparable to or even better than some catalysts composed of noble metals, such as titania-modified silver (0.47 eV) [48], and osmium and ruthenium atom doped graphene (0.52 eV) [26]. The Mo-doped BN monolayer presents even better performance for selective CO_2_ reduction than these catalysts. Although iridium-doped TiC applied as an electrocatalyst to selectively convert CO_2_ to CH_4_ is among the best values reported in the literature (limiting potential of −0.09 V [51]), iridium as a noble metal that comes at a high cost and has limits to its practical application. Above all, molybdenum as a non-noble-metal doped on BN monolayers presents both great performance in this theoretical study and is practically feasible for future experimental research.

Although the energy input for the last step is possibly somewhat high, it could be reduced by increasing the coverage of *OH based on the previous study [48]. Once H_2_O has been produced, CO_2_ can take its position, because CO_2_ adsorbed on Mo-doped BN monolayer is more thermodynamically stable than H_2_O, as mentioned in Figure 2. In summary, these results mean that the SAC, Mo-doped BN monolayer, can be reused for CRR. In addition, previous investigation has demonstrated that the interaction between Mo and the BN monolayer is very strong, and the catalyst has an excellent thermal stability for long-term use [45]. Overall, the study indicates that the Mo-doped BN monolayer has great potential for use as an efficient SAC for CRR.

### CRR mechanisms of the Mo-doped BN monolayer

**Figure 5:**
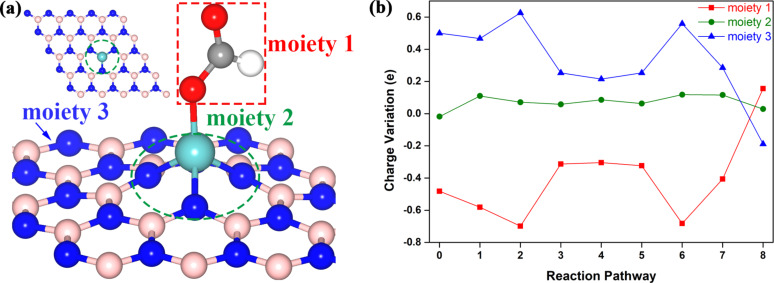
(a) Top and side view of three moieties divided with *OCHO. (b) Charge variation of the three moieties along the reaction pathway.

To further insight the catalytic performance of the Mo-doped BN monolayer for the selective CRR, we carried out the charge variation along the reaction pathway by Mulliken charge analysis. According to previous studies [45,52–53], especially the study of Mo-doped BN nanosheets as a catalyst for N_2_ fixation [45], the intermediate was divided into three moieties for research. These were the C*_x_*H*_y_*O*_z_* (moiety 1) as the product of CO_2_ hydrogenation reaction of each step, MoN_3_ (moiety 2) is the Mo atom and the three N atoms that the Mo atom linked with, and BN monolayer (moiety 3), as shown in Figure 5a. The variation of the charge distribution is illustrated in Figure 5b. The step 0 is the charge transfer to the adsorbed CO_2_. The CO_2_ molecule and the MoN_3_ gain 0.482 and 0.018 electrons from the BN monolayer, respectively. We can see from Figure 5b that the charge distributions of moiety 1 and moiety 3 obviously fluctuate with the following hydrogenation reduction steps. For instance, at the step 1, the moiety 1 (*OCHO) gains 0.581 electrons from both MoN_3_ and BN monolayers. In addition, there are about 0.467 electrons from the BN monolayer which contributes a large part to the total electron. In the same way, the BN monolayer contributes 0.626 electrons which occupy the vast majority of the electrons that moiety 1 (*OCH_2_O) gained at step 2. Along the whole reaction pathway, the role of the BN monolayer is to serve as an electron reservoir used for contributing or accepting electrons, while the charge fluctuation of MoN_3_ is smaller over the whole reaction as compared with previous reports [45,52]. In all, the MoN_3_ is the active site and can also be defined as a transmitter for charge transfer between the BN monolayer and moiety 1.

## Conclusion

In conclusion, we have systematically investigated TM atoms, including Sc to Zn, Mo, Ru, Rh, Pd and Ag, anchored on the boron-defective BN monolayer as an efficient SAC for CRR, as investigated by means of DFT calculations. The calculated results indicate that the Mo-doped BN monolayer exhibits remarkable electrocatalytic performance for the conversion of CO_2_ into hydrocarbon fuel. The limiting potential of the CO_2_ conversion to CH_4_ on the SAC, Mo-doped BN monolayer is relatively low with a value of −0.45 V, which is lower than most non-noble metal catalysts to selectively produce CH_4_. Therefore, the study demonstrates a potential electrocatalyst employing a non-noble metal with high catalytic efficiency for conversion CO_2_ into useful fuel under ambient conditions. This work provides important information at the atomic level for experimental researchers in search of low cost and efficient SACs for CO_2_ reduction.

## Computational Methods

All of the calculations were carried out by means of spin-polarized DFT with the DMol3 code [54–55]. The exchange and correlation potentials were calculated using the Perdew, Burke, and Ernzerhof (PBE) [56] functional within the generalized gradient approximation (GGA) [57]. The van der Waals (vdW) interactions were described using the empirical correction in Grimme’s scheme [58]. The calculational method has been successfully used for the investigation of selective adsorption and reaction of gases on BN nanomaterials [35,45,47]. The transition metal atoms were produced by density functional semi-core pseudopotential (DSPP), and the valence electronic structure is shown in Supporting Information File 1, Table S7, in which the core electrons are replaced by a single effective potential and the core is treated accurately by introducing some relativistic corrections [59]. The double-numeric polarized (DNP) basis was chosen as the basis set for other elements, which was set with a real-space cutoff at 5.0 Å. We used a conductor-like screening model (COSMO) to simulate a water solvent environment [60], and the dielectric constant was 78.54.

To construct the modes, we first built a periodic 5 × 5 BN supercell, whose vacuum region was 15 Å along the *z*-direction. The single TM atoms were doped at the boron vacancy sites [45]. All of the structures were completely optimized in a unit cell of 12.58 × 12.58 × 15.00 Å^3^ with convergence criterion of 1 × 10^−6^ a.u. for the energy and 0.005 Å for the displacements. For the 5 × 5 supercell, the k-point sampling of the Brillouin zone (BZ) adopted a 5 × 5 × 1 Monkhorst–Pack grid [61]. The Mulliken charge analysis was employed to calculate the charge distribution and transfer [62].

The whole reaction of CO_2_ reduction into hydrocarbon products involved eight elementary coupled proton and electron transfer (CPET) steps on the Mo-doped BN monolayer as follows:

[1] $${\text{CO}}_{2}+8{\text{H}}^{+}+8{\text{e}}^{-}\to {\text{CH}}_{4}+2{\text{H}}_{2}\text{O}$$

According to previous studies, a single metal atom performs as the active site for adsorption of gas and formation of carbon reduction intermediates. The hydrogen serves as the proton source (H_2_ ↔ 2(H^+^ + e^−^)) in the CO_2_ hydrogen evolution reaction of each CPET step [30,63]. The Gibbs free energy change (Δ*G*) is the relative energy of the total Gibbs free energy of the isolated CO_2_ molecule and the clean surface of the Mo-doped BN monolayer, which indicates the thermodynamic feasibly, and is determined as follows:

[2] $$\Delta G=\Delta E+\Delta ZPE-T\Delta S+\Delta G_{U}+\Delta G_{\text{pH}}$$

In this equation, Δ*E* represents the variation of the reaction energy obtained from DFT calculations as the expression: *∆E = E*_AB_ *− E*_A_ *− E*_B_, where Δ*ZPE* is zero-point energy (ZPE) difference between the products and reactants in the reaction whose expression is similar to Δ*E,* where *T* represents the temperature (*T* = 298.15 K), and Δ*S* represents the change of entropy. The entropies of free gas molecules and vibrational frequencies were all obtained from the NIST database [64], and the data of ZPE and entropy for the gas molecules at 298.15 K are shown in Table S1 in the Supporting Information File 1. Δ*G_U_* is the free energy of the electrode potential, which is Δ*G_U_* *= −n_e_U* (*n* is the number of electrons transferred corresponding to the elementary steps and *U* is the electrode potential). Δ*G*_pH_ is the free energy contribution of the H^+^ concentration. The expression is Δ*G*_pH_ *=* 2.303 × *k*_B_*T* × pH, where *k*_B_ is the Boltzmann constant (*k*_B_ = 1.38 × 10^−23^ J/K), and the pH was set at zero in the study to simulate acidic conditions.

## Supporting Information

File 1 A detailed description of the dataset.
