# Supplementary material for: Mo-doped boron nitride monolayer as a promising single-atom electrocatalyst for CO2 conversion
Source: Beilstein J Nanotechnol. 2019 Feb 22;10:540–8. doi: 10.3762/bjnano.10.55 (PMC6404464; doi:10.3762/bjnano.10.55)
Supplement: File 1 — A detailed description of the dataset. [file Beilstein_J_Nanotechnol-10-540-s001.pdf]

BEILSTEIN JOURNAL OF NANOTECHNOLOGY

Supporting Information

for

Mo-doped boron nitride monolayer as a promising single-atom electrocatalyst for CO_2 conversion

Qianyi Cui, Gangqiang Qin, Weihua Wang, Lixiang Sun, Aijun Du and Qiao Sun

Beilstein J. Nanotechnol. **2019,** *10,* 540–548. doi:10.3762/bjnano.10.55

A detailed description of the dataset

License and Terms: This is a supporting information file under the terms of the Creative Commons Attribution License (http://creativecommons.org/licenses/by/4.0). Please note that the reuse, redistribution and reproduction in particular requires that the authors and source are credited.
The license is subject to the *Beilstein Journal of Nanotechnology* terms and conditions: (https://www.beilstein-journals.org/bjnano)

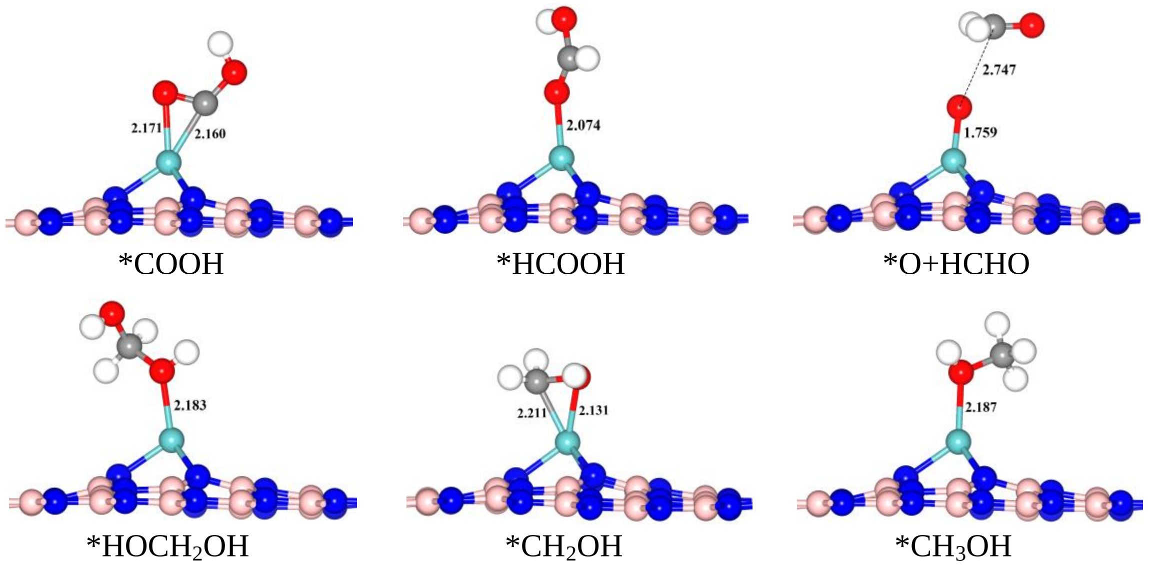

Figure S1: The configurations of the by-product in each step.

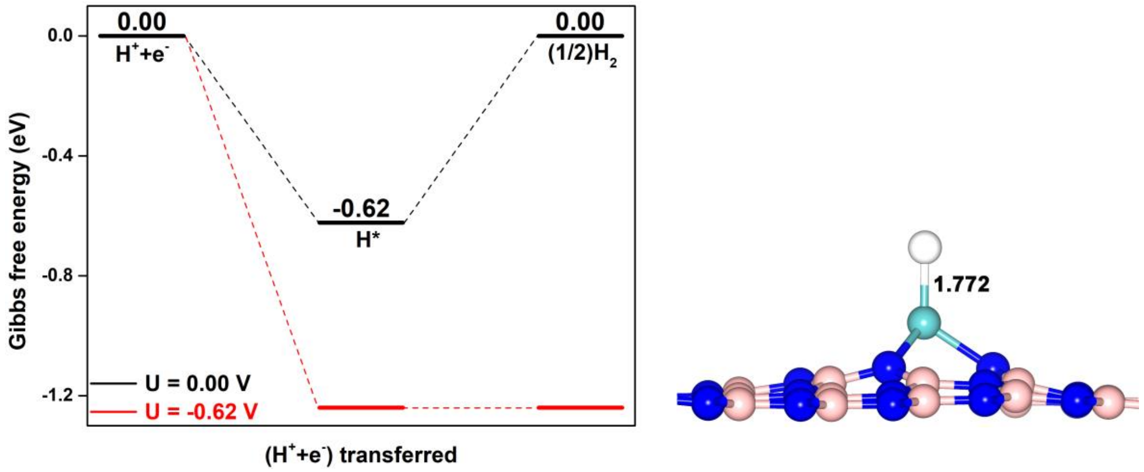

(a) (b)

Figure S2: Gibbs free energy diagram of the HER on the surface of Mo-doped BN monolayer (a) and the structure of the H^+/e^- adsorption (b). The bond length is labeled in Å. * represents chemisorbed species.

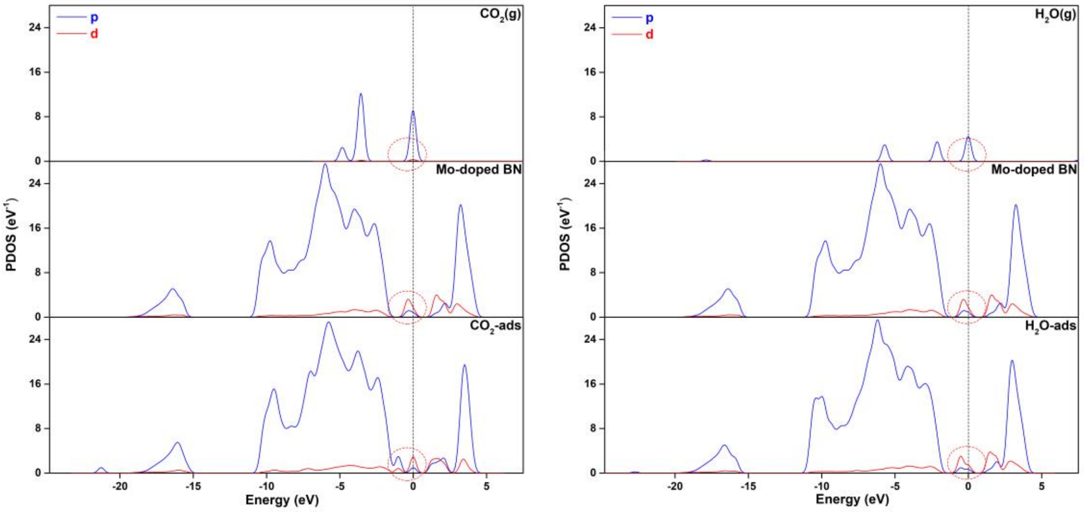

Figure S3: Projected density of states (PDOS) of the clean Mo-doped BN, free CO_2 and H_2O molecule and their adsorbed species on the surface of Mo-doped BN monolayer. The black dashed line setting at zero indicates the Fermi level and oval dotted line in red denotes overlapping peaks of p- and d-orbital around Fermi level.

.

Table S1: Thermodynamic parameters in eV, for the free H_2O, H_2, CO_2 and CH_4 gas molecules at 298.15 K and 101325 Pa [1].

Species	-TS	ZPE
H_2O (g)	-0.55	0.56
H_2 (g)	-0.39	0.27
CO_2 (g)	-0.64	0.27
CH_4 (g)	-1.20	1.20

The entropy (S) can be expressed as the sum of the translational, rotational, vibrational and electronic entropy. At fundamental electronic level: $S_e \approx 0$. So, the entropy calculation as the expression: $S = S_t + S_r + S_v$

Table S2: The values of adsorption energies in eV, corresponding to the isolated CO_2, H_2O and N_2 species on the TMs supported by the boron defective BN monolayer.

Metal	CO_2 E_{ads}	H_2O E_{ads}
Sc	-0.27	-0.83
Ti	-3.24	-3.68
V	-2.93	-3.30
Cr	-3.04	-3.58
Mn	-0.67	-1.43
Fe	-0.35	-0.96
Co	-0.27	-0.81
Ni	-0.34	-0.86
Cu	-1.36	-1.88
Zn	-0.38	-0.78
Mo	-1.09	-0.72
Ru	-0.41	-0.79
Rh	-0.33	-0.77
Pd	-0.28	-0.74
Ag	-0.24	-0.49

For the adsorption energies, $\Delta E_{ads} = E_{gas\text{–}catal} - E_{gas} - E_{catal}$, where $E_{gas\text{-}catal}$ represents the energy of the whole absorbed structure, E_{gas} represents the energy of free gas molecules and E_{catal} represents the energy of clean SACs surface, respectively.

Table S3: The properties of the most stable CO_2 absorbed structure includes proximal distance between CO_2 and TM atoms, O-C bond length in eV, O-C-O angle in ° and charge transfer of CO_2 in *e*.

Metal	CO_2-M distance (Å)	O-C-O Angle /°	O1-C bond length (Å)	O2-C bond length (Å)	Charge transfer (e)
Sc	2.350	179.8	1.177	1.166	0.073
Ti	1.975	130.6	1.299	1.214	-0.583
V	2.026	135.4	1.293	1.224	-0.461
Cr	1.959	141.2	1.284	1.205	-0.251
Mn	1.999	179.6	1.177	1.166	0.151
Fe	2.217	178.9	1.185	1.166	0.093
Co	2.309	178.8	1.182	1.168	0.076
Ni	2.229	179.0	1.180	1.168	0.089
Cu	2.373	179.8	1.176	1.170	0.070
Zn	2.436	179.5	1.183	1.168	0.067
Mo	2.092	135.7	1.309	1.215	-0.482
Ru	2.118	148.3	1.242	1.209	-0.243
Rh	2.399	179.3	1.179	1.169	0.092
Pd	2.346	179.2	1.181	1.168	0.102
Ag	2.646	179.9	1.176	1.171	0.056

Table S4: The properties of the most stable H_2O absorbed structure includes proximal distance between H_2O and TM atoms, H-O-H bond length in eV, H-O-H angle in ° and charge transfer of H_2O in *e*.

Metal	H_2O-M distance (Å)	H-O-H Angle /°	H1-O bond length (Å)	H2-O bond length (Å)	Charge transfer (e)
Sc	2.245	105.40	0.976	0.976	0.140
Ti	2.143	105.77	0.976	0.976	0.171
V	2.088	105.78	0.977	0.977	0.202
Cr	2.140	105.45	0.976	0.976	0.195
Mn	1.982	105.45	0.978	0.979	0.256
Fe	2.031	105.81	0.977	0.977	0.222
Co	2.035	105.43	0.978	0.978	0.235
Ni	2.012	105.63	0.978	0.978	0.236
Cu	2.096	104.81	0.977	0.976	0.205
Zn	2.081	104.73	0.977	0.977	0.217
Mo	2.332	104.67	0.976	0.976	0.156
Ru	2.220	105.11	0.977	0.977	0.198
Rh	2.228	104.28	0.977	0.977	0.209
Pd	2.208	104.43	0.978	0.978	0.228
Ag	2.284	104.25	0.976	0.976	0.192

Table S5: Gibbs free energy variation at 298.15 K in eV whose ZPE correction has been applied, and charge transfer in e, corresponding to CO_2 and different intermediates along the CO_2 reduction pathway by the Mo-doped BN monolayer. * refer to chemisorbed species. The total Gibbs free energy of the isolated CO_2 molecule and Mo-doped BN monolayer is defined as the zero.

species	Gibbs free energy variation (eV)	Charge transfer (*e*)
*CO_2	-0.83	-0.482
*OCHO	-1.35	-0.581
*COOH	-0.52	-0.251
*OCH_2O	-1.64	-0.699
*HCOOH	-0.63	0.003
*O+HCHO	-0.79	-0.026
*$HOCH_2O$	-2.17	-0.313
*CH_2O	-1.89	-0.304
*$HOCH_2OH$	-0.68	0.193
*CH_3O	-2.20	-0.323
*CH_2OH	-1.05	0.014
*CH_3OH	-1.42	0.196
*O	-2.64	-0.682
*OH	-2.74	-0.406
*H_2O	-2.29	-0.174

Table S6: Variation of Mulliken charge of the three moieties (shown in Fig. 5b) of the catalyst.

Reaction step	Moiety 1	Moiety 2	Moiety 3
0	-0.482	-0.018	0.500
1	-0.581	0.110	0.467
2	-0.699	0.071	0.626
3	-0.313	0.058	0.253
4	-0.304	0.086	0.215
5	-0.323	0.063	0.253
6	-0.682	0.118	0.559
7	-0.406	0.116	0.286
8	0.156	0.029	-0.188

Table S7: The valence electronic configurations of the transition metal by method of semi-core pseudopotentials (DSPP).

Element	Valence electronic structure	Element	Valence electronic structure
Sc	$3s^2 3p^6 3d^1 4s^2$	Cu	$3s^2 3p^6 3d^{10} 4s^1$
Ti	$3s^2 3p^6 3d^2 4s^2$	Zn	$3s^2 3p^6 3d^{10} 4s^2$
V	$3s^2 3p^6 3d^3 4s^2$	Mo	$4s^2 4p^6 4d^5 5s^1$
Cr	$3s^2 3p^6 3d^5 4s^1$	Ru	$4s^2 4p^6 4d^6 5s^2$
Mn	$3s^2 3p^6 3d^5 4s^2$	Rh	$4s^2 4p^6 4d^7 5s^2$
Fe	$3s^2 3p^6 3d^6 4s^2$	Pd	$4s^2 4p^6 4d^8 5s^2$
Co	$3s^2 3p^6 3d^7 4s^2$	Ag	$4s^2 4p^6 4d^{10} 5s^1$
Ni	$3s^2 3p^6 3d^8 4s^2$		

References

[1] Computational chemistry comparison and benchmark database. Https://cccbdb.Nist.Gov/, in, 2005.
